# Near-Infrared Photoactivatable Immunomodulatory Nanoparticles for Combinational Immunotherapy of Cancer

**DOI:** 10.3389/fchem.2021.701427

**Published:** 2021-05-24

**Authors:** Ningyue Yu, Mengbin Ding, Jingchao Li

**Affiliations:** Shanghai Engineering Research Center of Nano-Biomaterials and Regenerative Medicine, College of Chemistry, Chemical Engineering and Biotechnology, Donghua University, Shanghai, China

**Keywords:** near-infrared light, nanomedicine, immunotherapy, photoactivation, phototherapy

## Abstract

As a promising treatment option for cancer, immunotherapy can eliminate local and distant metastatic tumors and even prevent recurrence through boosting the body’s immune system. However, immunotherapy often encounters the issues of limited therapeutic efficacy and severe immune-related adverse events in clinical practices, which should be mainly due to the non-specific accumulations of immunotherapeutic agents. Activatable immunomodulatory agents that are responsive to endogenous stimuli in tumor microenvironment can afford controlled immunotherapeutic actions, while they still face certain extent of off-target activation. Since light has the advantages of noninvasiveness, simple controllability and high spatio-temporal selectivity, therapeutic agents that can be activated by light, particularly near-infrared (NIR) light with minimal phototoxicity and strong tissue penetrating ability have been programmed for cancer treatment. In this mini review, we summarize the recent progress of NIR photoactivatable immunomodulatory nanoparticles for combinational cancer immunotherapy. The rational designs, constructions and working mechanisms of NIR photoactivatable agents are first briefly introduced. The uses of immunomodulatory nanoparticles with controlled immunotherapeutic actions upon NIR photoactivation for photothermal and photodynamic combinational immunotherapy of cancer are then summarized. A conclusion and discussion of the existing challenges and further perspectives for the development and clinical translation of NIR photoactivatable immunomodulatory nanoparticles are finally given.

## Introduction

Immunotherapy is a type of cancer treatment that uses the body’s immune system to fight cancer (Del Paggio, 2018). Different from traditional treatment methods such as surgery, chemotherapy and radiotherapy that can only be effective for local tumors, immunotherapy is able to remove both local and distant metastatic tumors, and even prevent tumor recurrence (Nam et al., 2019; Li et al., 2021). Immune checkpoint blockade therapy, cancer vaccines, and adoptive T cell therapy are three key approaches for cancer immunotherapy (Pardoll, 2012; Kuai et al., 2017; Fraietta et al., 2018). With the approval of different immunotherapeutic agents by the U.S. Food and Drug Administration (FDA), a great success has been achieved for cancer immunotherapy in the clinic (Zhang and Pu, 2020). However, these agents after traditional systemic administration often have non-specific accumulations in normal tissues, resulting in limited therapeutic efficacy and severe immune-related adverse events (irAEs), such as diabetes mellitus, thyroid dysfunction, hypophysitis, myocarditis, and hypokalemia (Boutros et al., 2016; Song et al., 2018b; Chiang et al., 2018; Eun et al., 2019). Nanomedicines can modulate pharmacological profiles of immunotherapeutic agents and integrate additional therapeutic modalities to enhance the antitumor immunity, while these critical concerns of cancer immunotherapy have not been addressed because of uncontrolled release of agents from nanoparticles (Caster et al., 2019; Lim et al., 2019).

Development of smart immunotherapeutic nanoagents with controlled activation is a promising strategy to improve the efficacy and safety of cancer immunotherapy (Li and Pu, 2020; Wang et al., 2021). Activatable immunotherapeutic nanoagents may be specifically responsive to endogenous stimuli in tumor microenvironment, such as lower pH, higher glutathione concentrations, higher reactive oxygen species (ROS) levels, increased expression levels of certain enzymes, and hypoxic conditions to achieve controlled antitumor immunity (Cheng et al., 2018; Im et al., 2018; Chen et al., 2019; Peng et al., 2019; Wang et al., 2019). However, these endogenous stimuli also exist in normal tissues, which often results in off-target immune activation for such immunotherapeutic nanoagents (Tang et al., 2018).

As an external stimulation, light has the advantages of noninvasiveness, good controllability, and high spatio-temporal selectivity, and thus has been widely utilized for photoactivation of therapeutic agents (Karimi et al., 2017). The photoactivatable agents can be formed via incorporating therapeutic molecules with light-sensitive moieties, while most of them only respond to ultraviolet and visible light (100–650 nm) that shows shallow tissue-penetration depth and high phototoxicity (Gnanasammandhan et al., 2016). By contrast, near-infrared (NIR) light (650–1,700 nm) has minimal photodamage and strong penetrating capability in biological tissues, and thus has been employed to regulate the pharmacological activities of immunotherapeutic agents with the development of NIR photoactivatable immunomodulatory nanoparticles (Lin et al., 2017). Moreover, NIR light can be used for photothermal therapy (PTT) and photodynamic therapy (PDT) of tumors in the presence of photothermal agents or photosensitizers, which will contribute to enhanced antitumor immunity (Tao et al., 2014; Yang et al., 2020). Thus, NIR photoactivatable immunomodulatory nanoparticles with controlled activity and multiple therapeutic actions have been developed for combinational immunotherapy of cancer.

We herein summarize recent progress of NIR photoactivatable immunomodulatory nanoparticles for combinational immunotherapy of cancer. Design principles and construction approaches of NIR photoactivatable immunomodulatory nanoparticles and their working mechanisms to achieve regional selectivity of immune activation are briefly summarized. The feasibility of such NIR photoactivatable immunomodulatory nanoparticles for PTT/PDT combinational immunotherapy to obtain an enhanced therapeutic efficacy and biosafety is highlighted with some discussions of their potential concerns. A general conclusion and discussion of existing challenges and further perspectives in this field are then given.

## Design of Near-Infrared Photoactivatable Immunomodulatory Nanoparticles

NIR light can be converted into local heat and singlet oxygen (^1^O_2_) by photothermal agents and photosensitizers, respectively, which will allow for on-demand release and/or activation of therapeutic agents (Li et al., 2019a; Li et al., 2019b; Uthaman et al., 2020). In view of the two different mechanisms, immunomodulatory nanoparticles with photothermal and photodynamic activatable pharmacological actions have been designed and constructed for PTT and PDT combinational immunotherapy.

Photothermal activatable immunomodulatory nanoparticles are generally formed via integrating thermal-responsive components into nanoparticles containing immunodrugs and photothermal agents. Upon NIR laser irradiation, photothermal agents generate local heat to destroy the thermal-responsive components for controlled release of immunotherapeutic agents from nanoparticles. As such, these photothermal activatable immunomodulatory nanoparticles can afford a synergetic action of PTT and immunotherapy. The fabrications of photodynamic activatable immunomodulatory nanoparticles rely on the uses of ^1^O_2_-cleavable linkers to conjugate small molecular agents with photosensitizer-based nanoparticles. During PDT processes with NIR laser irradiation, ^1^O_2_ is generated by photosensitizers to initiate the cleavage of ^1^O_2_-cleavable linkers to liberate caged agents for *in-situ* activation. Such controlled activation of immunotherapeutic agents in combination with PDT will potentiate antitumor immunity.

## Photothermal Activatable Immunomodulatory Nanoparticles

Due to the generation of heat during PTT, thermal-responsive phase change materials have been widely used to construct photothermal drug delivery systems to achieve on-demand release of therapeutic agents (Bordat et al., 2019). As an example, Yang’s group reported a phase-transformation nanoparticle to facilitate delivery of anti-PD1 antibody (aPD1) for PTT synergized immunotherapy of melanoma (Zhang et al., 2019). They encapsulated aPD1, iron oxide and perfluoropentane (PFP) into poly(lactic-co-glycolic acid) (PLGA) shell modified with PEG and Gly-Arg-Gly-Asp-Ser (GRGDS) peptides to form phase-transformation nanoparticles (GOP@aPD1). PFP is a type of phase change material that converts from liquid to gas after the temperature increase. Due to the enhanced permeability and retention (EPR) effect and active targeting of GRGDS peptides, GOP@aPD1 showed an effective accumulation at the melanoma sites after systemic administration. Iron oxide nanoparticles acted as a photothermal agent to generate heat under 660 nm laser irradiation, which resulted in phase-transformation of PFP to destroy nanoparticles for controlled release of aPD1. Such synergistic action of PTT and aPD1-mediated immunotherapy achieved enhanced antitumor efficacy, greatly inhibiting the growth of B16F10 tumors. In view of their high biosafety and biocompatibility, GOP@aPD1 was promising for clinical translation, while the long-term biodegradation of iron oxide should be considered.

Thermo-responsive liposomes have shown a great promise for on-demand delivery of drugs for cancer therapy (Zhen et al., 2018; Dai et al., 2019). Jin and coworkers developed a thermo-responsive liposome containing indocyanine green (ICG) as the photothermal agents and polyinosinic:polycytidylic acid (poly I:C) as the immune stimulatory molecule for photothermal-immunotherapy (Xu et al., 2019). Due to the photothermal effect of ICG, NIR laser irradiation at 808 nm increased the temperature to destroy thermo-responsive liposome to release poly I:C. The PTT effect induced cancer cell death and ablation of tumors, and the released poly I:C promoted the maturation of dendritic cells (DCs), which synergistically triggered an antigen-specific immune response to prevent lung metastasis following intravenous injection of cancer cells. Most of the agents in such photothermal activatable immunomodulatory nanoparticles have been approved by FDA, which greatly promotes their clinical uses, while the photostability of ICG should be improved.

NIR photothermal activatable immunomodulatory nanoparticles have shown great promising for cancer immunotherapy, while the tissue penetration depth of first NIR light (NIR-I) was still limited (< 1 cm) (Mura et al., 2013; Jiang et al., 2020). In contrast, the second NIR (NIR-II) light had an increased tissue penetration depth as high as 3–5 cm, which allowed the construction of NIR-II photothermal activatable immunomodulatory nanoparticles (Lyu et al., 2019). As an example, Pu’s group recently constructed a semiconducting polymer nanoadjuvant (SPN_II_R) with NIR-II photothermally induced on-demand release of immune adjuvants for enhanced cancer immunotherapy (Li et al., 2020a). Semiconducting polymer-based nanoparticles can be used for molecular imaging, phototherapy, and photoregulation because of their good biocompatibility and unique optical property (Jiang and Pu, 2018). Such SPN_II_R with a core-shell structure contained three main components: a semiconducting polymer nanoparticle core as the NIR-II photothermal converter, a toll-like receptor (TLR) agonist (R848) as the immunotherapeutic adjuvant, and a thermally responsive lipid shell on the nanoparticle surface. The thermally responsive lipid shell was constructed using 1,2-dipalmitoyl-sn-glycero-3-phosphocholine (DPPC), a thermal phospholipid with a phase-transition temperature of 41°C, and an amphiphilic polymer, 1,2-distearoyl-sn-glycero-3-phosphoethanolamine-poly(ethylene glycol) (DSPE-PEG), and could be melted after a photothermal effect. SPN_II_R exhibited an effective accumulation into tumors after systemic administration, which may be due to their small size and surface PEG corona. Upon NIR-II photoactivation at 1,064 nm, SPN_II_R produced heat to induce tumor cell death and immunogenic cell death (ICD). Meanwhile, the generated heat could melt thermally responsive lipid shell for controlled release of R848 to active TLR7/TLR8, thereby promoting the maturation of DCs and priming of antitumor T cells. Via the synergetic action of NIR-II PTT and immunotherapy, the growth of both primary and distant tumors and lung metastasis in a 4T1 tumor-bearing BALB/c mouse model was effectively suppressed after a single treatment. Although these promising results, the stability of SPN_II_R in biological systems should be a concern since the phospholipid could be easily oxidized and hydrolyzed. In addition, the doping of imaging probes was required to monitor the accumulation of nanoparticles into tumors so that to optimize the therapeutic window.

**FIGURE 1:**
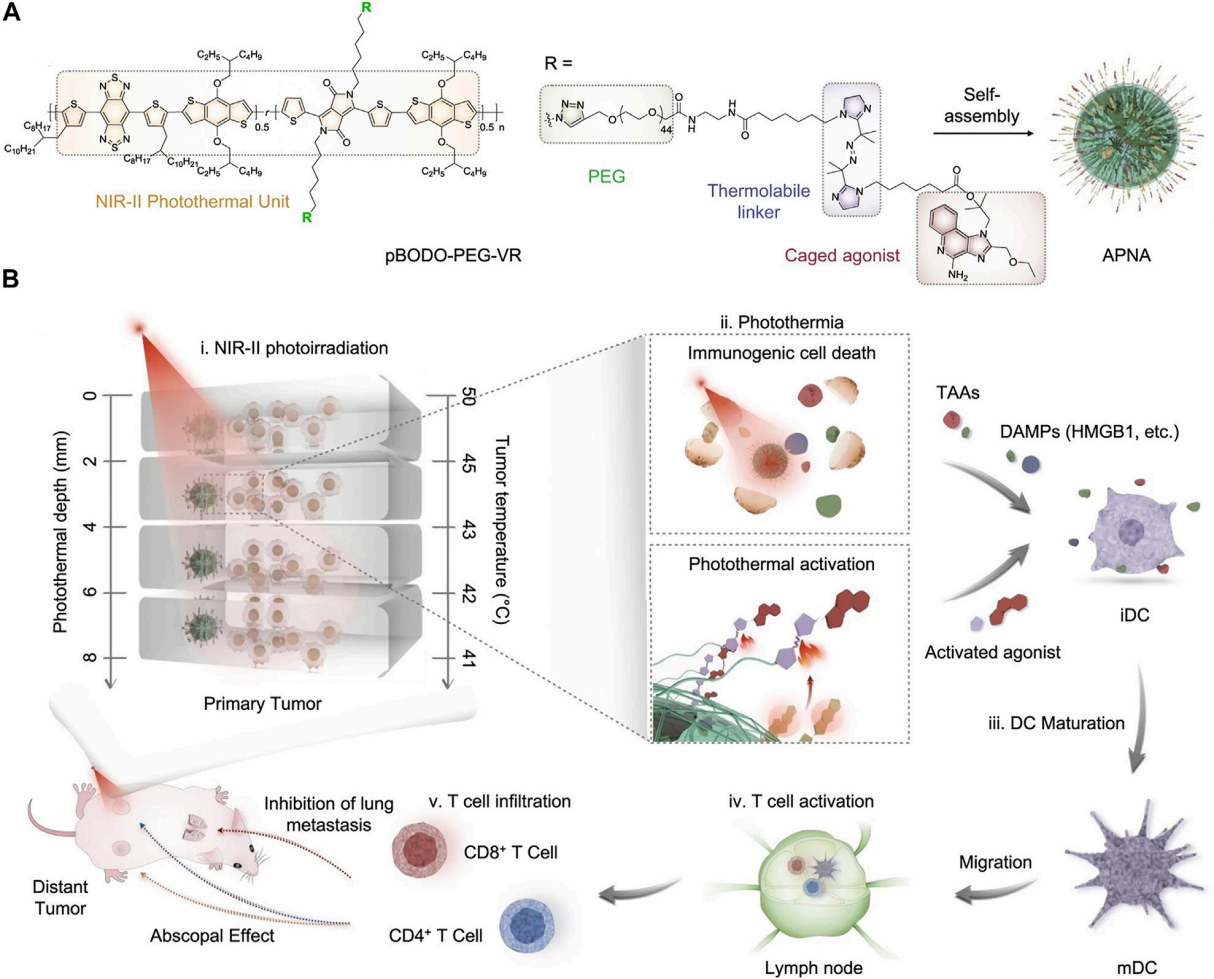
**(A)** Chemical structure of NIR-II absorbing semiconducting polymer conjugated with R848 through a thermo-responsive linker and self-assembly of APNA. **(B)** Mechanism of antitumor immune response by APNA-mediated NIR-II photothermal immunotherapy. Reproduced, with permission, from Jiang et al. (2021). Copyright 2021, Nature Publishing Group.

In addition to thermally responsive phospholipid, thermo-responsive linker has been utilized to construct NIR-II photothermal activatable drug delivery nanosystems for cancer therapy (Lei et al., 2016). In a recent study of Pu’s group, the authors reported a photothermally activatable polymeric pronanoagonist (APNA) with NIR-II light regulated activity for PTT combinational immunotherapy (Jiang et al., 2021). Within APNA, a NIR-II light-absorbing semiconducting polymer served as a photothermal transducer, which was conjugated with R848 through a thermo-responsive linker (Figure 1A). The APNA mediated photothermal effect under 1,064 nm laser irradiation, which not only led to direct photothermal ablation of tumors and elicitation of ICD of tumor cells, but also resulted in cleavage of the thermo-responsive linker to liberate caged R848 that converted into an intact form after a subsequent hydrolysis by esterase (Figure 1B). The maximal activation ratio of R848 was only 8.5% if the photothermal temperature was kept at 37°C under 1,064 nm laser irradiation, which was dramatically increased to 74% when the photothermal temperature was elevated to above 45°C. Such a photo-regulated immune activation could be achieved in primary tumors of 4T1 tumor-bearing mice at a depth of 8 mm, thereby enabling complete eradication of primary tumors and efficient inhibition of both distant tumors and lung and liver metastasis without causing obvious side effects. Moreover, the relationship between intratumoral immune activation and photothermal depth was unveiled. This work indeed provided a novel approach for remote spatiotemporal controlling of immune activation. A challenge was the complicated synthesis and fabrication processes that would limit the productivity of such immunomodulatory nanoparticles for clinical uses.

## Photodynamic Activatable Immunomodulatory Nanoparticles

The generation of ROS in the PDT process allowed the construction of photodynamic activatable nanoparticles for cancer therapy (Yang et al., 2016; Pei et al., 2018; Uthaman et al., 2020). For example, Yang and Song’s groups reported a black phosphorus quantum dots (BPQDs)-based NIR/ROS-responsive immunoadjuvant nanoparticles for cancer photodynamic immunotherapy (Li et al., 2020b). PEG and ROS-responsive poly(propylene sulfide) (PPS) conjugated with pyrene were grafted onto BPQDs, which was used to encapsulate cytosine-phosphate-guanine oligodeoxynucleotides (CpG ODNs) as a potent adjuvant to form nanoparticles via self-assembly. As an agonist for TLR9, CpG ODNs greatly promote DC maturation, proinflammatory cytokine and chemokine secretion, leading to the activation of immunity (Scheiermann and Klinman, 2014). Upon 660 nm laser irradiation, BPQDs produced ROS to trigger the transformation of hydrophobic PPS to hydrophilic ones, which resulted in nanoparticle disassembly to release CPG ODNs in tumor microenvironment to enhance immunity and small sized BPQDs that could penetrate in deep tumors. As such, the synergistic PDT immunotherapy effectively inhibited the growth of 4T1 tumors and suppressed distant tumor growth and metastasis. In addition to antitumor effect, the on-demand dissociation of nanoparticles to release BPQDs increased tumor penetration for enhanced photoacoustic (PA) imaging, providing a stimuli-responsive nanoplatforms for cancer theranostics, which was much different from most of photoactivatable immunomodulatory nanoparticles.

Sun and coworkers recently reported a sorafenib and chlorin e6 (Ce6) co-loaded ROS-responsive nanoparticle to improve antitumor responses for PDT combinational immunotherapy (Sun et al., 2020). Sorafenib is a small molecular multi-kinase inhibitor that inhibits the functions of immunosuppressive cells in tumor microenvironment, resulting in augmented antitumor immunity via enhancing the activities of tumor-specific cytotoxic T cells (Chen et al., 2014). A ROS-responsive PEGylated hyperbranched polyphosphate containing ^1^O_2_-cleavable thioketal linkers was utilized to simultaneously encapsulate Ce6 as a hydrophobic NIR photosensitizer and sorafenib, forming photodynamic activatable immunomodulatory nanoparticles. Under 660 nm laser irradiation, Ce6 generated ^1^O_2_ to cleave the ^1^O_2_-responsive linkers to destruct the nanoparticles, leading to on-demand release of sorafenib in tumor sites. After treatment in 4T1 tumor-bearing mice, the synergistical action of low-dose PDT and rapidly released sorafenib modulated the immunosuppressive tumor microenvironment to trigger a strong T cell-dependent antitumor immunity, suppressing the growth of primary and distant tumors. Although this was a promising way to achieve controlled release of drugs for amplified antitumor effects, the penetration depth of 660 nm laser was limited.

**FIGURE 2:**
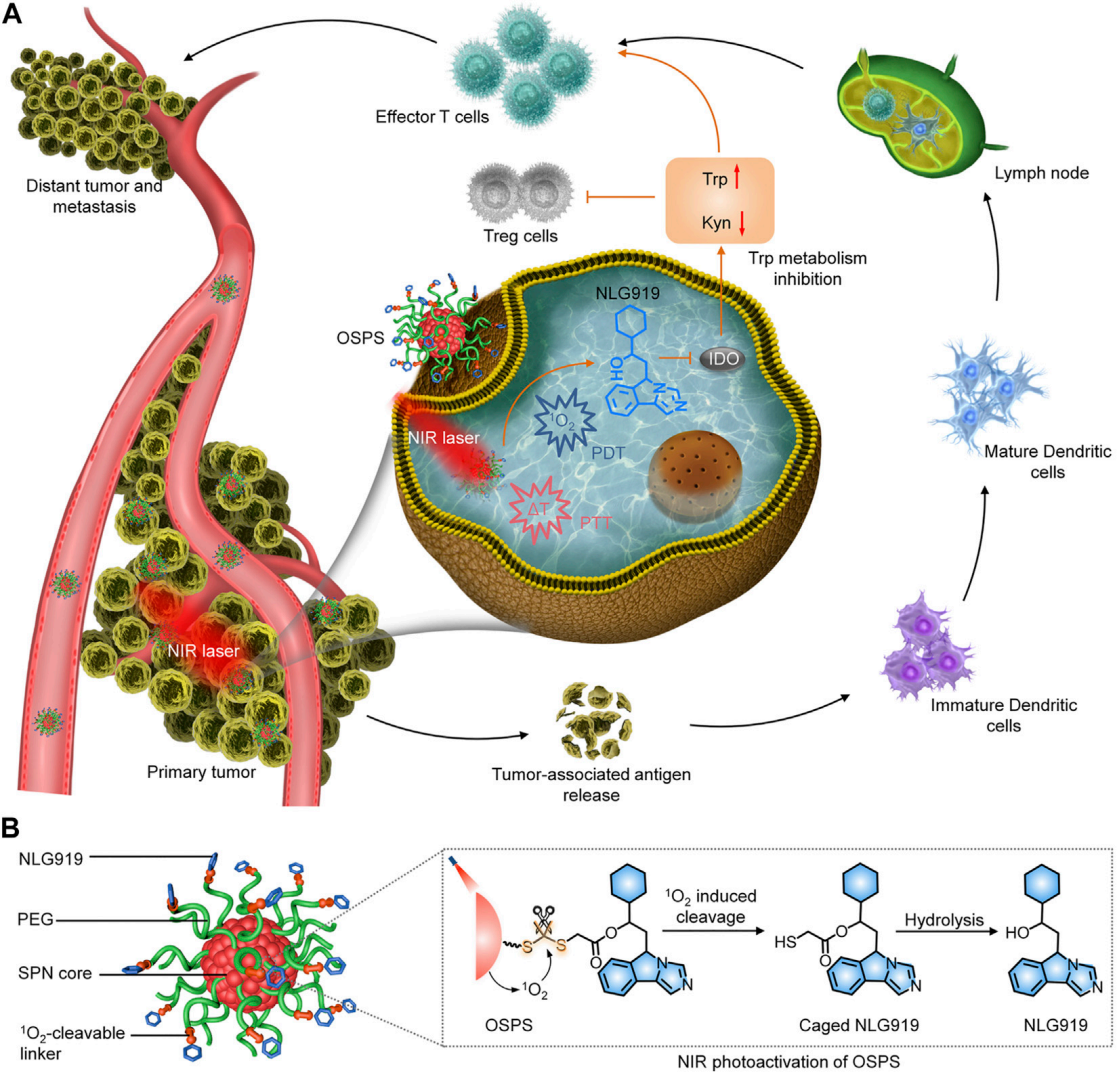
**(A)** Photoactivation of OSPS for synergistic therapeutic action including phototherapy and checkpoint blockade immunotherapy. **(B)** Structure and NIR photoactivation mechanism of OSPS. Reproduced, with permission, from Li et al. (2019a). Copyright 2019, John Wiley & Sons Ltd.

Li and coworkers developed an organic semiconducting pro-nanostimulant (OSPS) with a NIR photoactivatable immunotherapeutic action for cancer immunotherapy (Figure 2A) (Li et al., 2019c). OSPS was formed through conjugating a photodynamic semiconducting polymer with an indoleamine 2,3-dioxygenase (IDO) inhibitor (NLG919) via a ^1^O_2_-responsive linker, followed by self-assembly. The overexpressed IDO enzyme by most cancers degraded the essential amino acid tryptophan (Trp) into kynurenine (Kyn), resulting in immunosuppressive tumor microenvironment (Feng et al., 2018). Owing to its small size and stealthy PEG surface corona, OSPS showed an effective accumulation in subcutaneous tumors after systemic administration, in which, OSPS produced heat and ^1^O_2_ upon 808 nm laser irradiation for combinational PTT and PDT, and generation of tumor-associated antigens (TAAs). Meanwhile, the produced ^1^O_2_ specifically triggered the cleavage of ^1^O_2_-responsive linkers to release caged NLG919, which would undergo a rapid hydrolysis to generate active NLG919 (Figure 2B). Such a remote photoactivation of immunostimulants contributed to modulation of immunosuppressive tumor microenvironment, which in combination with OSPS-mediated phototherapy triggered a strong antitumor immune response. In a 4T1 tumor-bearing mouse model, a single treatment of OSPS could effectively inhibited the growth of both primary and distant tumors and prevented lung metastasis. If the drug loading capability can be further improved, this photoactivatable organic pro-nanostimulants should have a promise for more effective and safer immunotherapy.

Using the ^1^O_2_-responsive linker, Pu’s group reported an organic polymer nanoenzyme (SPNK) with a NIR photoactivatable enzymatic activity for photodynamic immunometabolic therapy (Zeng et al., 2021). SPNK was constructed based on a semiconducting polymer nanoparticle as the photosensitizer, which was conjugated with kynureninase (KYNase) via PEG chain containing ^1^O_2_-responsive linker. KYNase is an enzyme that catalyzes the degradation of immunosuppressive Kyn in IDO overexpressed tumors to reverse Kyn-induced immune suppression (Triplett et al., 2018). Such a photoactivation of nanoenzyme could significantly promote the proliferation and infiltration of effector T cells into B16F10 tumors in living mice, thus triggering an enhanced antitumor T cell immunity. As such, SPNK-mediated synergistic therapeutic action led to complete inhibition of primary tumors and effective suppression of distant tumors. This study provided a novel photodynamic approach for remote control of enzymatic activity to modulate immunometabolism for cancer immunotherapy. However, the synthesis conditions need to be strictly controlled to keep the activity of enzymes, and some strategies should be used to prevent degradation of enzymes by hydrolases during blood circulation.

The activation of abovementioned immunomodulatory nanoparticles relies on the generated ^1^O_2_. As PDT can induces cell death, apoptosis-related biomarkers responsive nanoparticles have also been developed for cancer therapy. Zhang’s group reported a peptide self-assembled nanoparticle for PDT combinational immunotherapy (Song et al., 2018a). The peptide consisted of palmitic acid and protoporphyrin IX (PpIX) as the hydrophobic part and a hydrophilic PEG conjugated with an IDO inhibitor 1-methyltryptophan (1MT) through a caspase-responsive peptide sequence, Asp-Glu-Val-Asp (DEVD). The peptide could self-assemble into nanoparticles that effectively accumulated into tumor tissues. Upon 630 nm laser irradiation, the peptide nanoparticles produced ^1^O_2_ via PDT effect to induce apoptosis of cancer cells, leading to caspase-3 expression and formation of TAAs, which facilitated antitumor immune response. The expressed caspase-3 could cleave DEVD peptide sequence to release 1MT to further strengthen the immunity and activate CD8^+^ T cells. Such synergistic effect of PDT and immunotherapy mediated by peptide nanoparticles effectively inhibited the growth of primary tumors and lung metastasis in a CT26 tumor-bearing mouse model. This study provided a unique strategy to regulate the delivery of immunotherapeutic agents with a very high efficacy, while the tissue penetration of light source needs to be further improved for their applications of large and deep-seated tumors.

Controlled antitumor immunity have been achieved by photodynamic activatable nanoparticles through regulating the activation of immunotherapeutic agents, while they still have some limitations. The endogenous ROS existed in the biological systems potentially results in off-target activation of drugs and thereby induces side effects. The biosafety of products for ROS-responsive moieties after photoactivation is questionable and needs to be systematically investigated. In addition, all existing photodynamic activatable nanoparticles only respond to visible or NIR-I light, and those that can be activated by NIR-II light have not been explored.

## Discussion

We herein have summarized the recent development of immunomodulatory nanoparticles with NIR photoactivatable pharmacological features for combinational immunotherapy of cancer. Due to the existences of photothermal agents or photosensitizers, these immunomodulatory nanoparticles can generate heat or ROS upon NIR photoirradiation to destroy thermal- or ROS-responsive moieties to allow on-demand release of immunotherapeutic agents, which greatly improve the bioavailability and selectivity. By combining with PTT and/or PDT, such NIR photoactivatable antitumor immunity often leads to improved therapeutic efficacy in treating tumors and preventing metastases.

Before the successful translation of NIR photoactivatable immunomodulatory nanoparticles for clinical trials, some key issues should be addressed. First, the long-term biosafety of nanoparticles in living bodies is questionable and should be systemically evaluated. Development of biodegradable or clearable nanoparticles is a good way to address this issue (Li and Pu, 2019). Second, the tissue penetration depths of NIR-I and NIR-II light are still limited and only suitable for superficial tumors. The use of light delivery technologies can achieve deep delivery of NIR light into biological tissues for treatment of deep-seated tumors (Maruoka et al., 2018). Third, it is challenging for real-time monitoring of dynamic immune activation to evaluate therapeutic efficacy. Imaging reporters that are specifically responsive to immune response can be combined with immunomodulatory nanoparticles to realize immune theranostics of cancer (He et al., 2020; Ramesh et al., 2020). In the future, these NIR photoactivatable immunomodulatory nanoparticles may show a great promise for clinical treatment of cancer and other diseases, such as infectious diseases and autoimmunity.

## References

[B1] Bordat A. Boissenot T. Nicolas J. Tsapis N. (2019). Thermoresponsive Polymer Nanocarriers for Biomedical Applications. Adv. Drug Deliv. Rev. 138, 167–192. 10.1016/j.addr.2018.10.005 30315832

[B2] Boutros C. Tarhini A. Routier E. Lambotte O. Ladurie F. L. Carbonnel F. (2016). Safety Profiles of Anti-CTLA-4 and Anti-PD-1 Antibodies Alone and in Combination. Nat. Rev. Clin. Oncol. 13, 473–486. 10.1038/nrclinonc.2016.58 27141885

[B3] Caster J. M. Callaghan C. Seyedin S. N. Henderson K. Sun B. Wang A. Z. (2019). Optimizing Advances in Nanoparticle Delivery for Cancer Immunotherapy. Adv. Drug Deliv. Rev. 144, 3–15. 10.1016/j.addr.2019.07.009 31330165 PMC11849717

[B4] Chen M.-L. Yan B.-S. Lu W.-C. Chen M.-H. Yu S.-L. Yang P.-C. (2014). Sorafenib Relieves Cell-Intrinsic and Cell-Extrinsic Inhibitions of Effector T Cells in Tumor Microenvironment to Augment Antitumor Immunity. Int. J. Cancer 134, 319–331. 10.1002/ijc.28362 23818246

[B5] Chen Q. Chen G. Chen J. Shen J. Zhang X. Wang J. (2019). Bioresponsive Protein Complex of aPD1 and aCD47 Antibodies for Enhanced Immunotherapy. Nano Lett. 19, 4879–4889. 10.1021/acs.nanolett.9b00584 31294571

[B6] Cheng K. Ding Y. Zhao Y. Ye S. Zhao X. Zhang Y. (2018). Sequentially Responsive Therapeutic Peptide Assembling Nanoparticles for Dual-Targeted Cancer Immunotherapy. Nano Lett. 18, 3250–3258. 10.1021/acs.nanolett.8b01071 29683683

[B7] Chiang C.-S. Lin Y.-J. Lee R. Lai Y.-H. Cheng H.-W. Hsieh C.-H. (2018). Combination of Fucoidan-Based Magnetic Nanoparticles and Immunomodulators Enhances Tumour-Localized Immunotherapy. Nat. Nanotech 13, 746–754. 10.1038/s41565-018-0146-7 29760523

[B8] Dai Y. Su J. Wu K. Ma W. Wang B. Li M. (2019). Multifunctional Thermosensitive Liposomes Based on Natural Phase-Change Material: Near-Infrared Light-Triggered Drug Release and Multimodal Imaging-Guided Cancer Combination Therapy. ACS Appl. Mater. Inter. 11, 10540–10553. 10.1021/acsami.8b22748 30807086

[B9] Del Paggio J. C. (2018). Cancer Immunotherapy and the Value of Cure. Nat. Rev. Clin. Oncol. 15, 268–270. 10.1038/nrclinonc.2018.27 29459643

[B10] Eun Y. Kim I. Y. Sun J.-M. Lee J. Cha H.-S. Koh E.-M. (2019). Risk Factors for Immune-Related Adverse Events Associated with Anti-PD-1 Pembrolizumab. Sci. Rep. 9, 14039. 10.1038/s41598-019-50574-6 31575933 PMC6773778

[B11] Feng B. Zhou F. Hou B. Wang D. Wang T. Fu Y. (2018). Binary Cooperative Prodrug Nanoparticles Improve Immunotherapy by Synergistically Modulating Immune Tumor Microenvironment. Adv. Mater. 30, 1803001. 10.1002/adma.201803001 30063262

[B12] Fraietta J. A. Lacey S. F. Orlando E. J. Pruteanu-Malinici I. Gohil M. Lundh S. (2018). Determinants of Response and Resistance to CD19 Chimeric Antigen Receptor (CAR) T Cell Therapy of Chronic Lymphocytic Leukemia. Nat. Med. 24, 563–571. 10.1038/s41591-018-0010-1 29713085 PMC6117613

[B13] Gnanasammandhan M. K. Idris N. M. Bansal A. Huang K. Zhang Y. (2016). Near-IR Photoactivation Using Mesoporous Silica-Coated NaYF4:Yb,Er/Tm Upconversion Nanoparticles. Nat. Protoc. 11, 688–713. 10.1038/nprot.2016.035 26963631

[B14] He S. Li J. Lyu Y. Huang J. Pu K. (2020). Near-infrared Fluorescent Macromolecular Reporters for Real-Time Imaging and Urinalysis of Cancer Immunotherapy. J. Am. Chem. Soc. 142, 7075–7082. 10.1021/jacs.0c00659 32196318

[B15] Im S. Lee J. Park D. Park A. Kim Y.-M. Kim W. J. (2018). Hypoxia-triggered Transforming Immunomodulator for Cancer Immunotherapy via Photodynamically Enhanced Antigen Presentation of Dendritic Cell. ACS. Nano 13, 476–488. 10.1021/acsnano.8b07045 30563320

[B16] Jiang Y. Huang J. Xu C. Pu K. (2021). Activatable Polymer Nanoagonist for Second Near-Infrared Photothermal Immunotherapy of Cancer. Nat. Commun. 12, 1–14. 10.1038/s41467-021-21047-0 33531498 PMC7854754

[B17] Jiang Y. Pu K. (2018). Multimodal Biophotonics of Semiconducting Polymer Nanoparticles. Acc. Chem. Res. 51, 1840–1849. 10.1021/acs.accounts.8b00242 30074381

[B18] Jiang Y. Zhao X. Huang J. Li J. Upputuri P. K. Sun H. (2020). Transformable Hybrid Semiconducting Polymer Nanozyme for Second Near-Infrared Photothermal Ferrotherapy. Nat. Commun. 11, 1857. 10.1038/s41467-020-15730-x 32312987 PMC7170847

[B19] Karimi M. Sahandi Zangabad P. Baghaee-Ravari S. Ghazadeh M. Mirshekari H. Hamblin M. R. (2017). Smart Nanostructures for Cargo Delivery: Uncaging and Activating by Light. J. Am. Chem. Soc. 139, 4584–4610. 10.1021/jacs.6b08313 28192672 PMC5475407

[B20] Kuai R. Ochyl L. J. Bahjat K. S. Schwendeman A. Moon J. J. (2017). Designer Vaccine Nanodiscs for Personalized Cancer Immunotherapy. Nat. Mater 16, 489–496. 10.1038/nmat4822 28024156 PMC5374005

[B21] Lei Q. Qiu W.-X. Hu J.-J. Cao P.-X. Zhu C.-H. Cheng H. (2016). Multifunctional Mesoporous Silica Nanoparticles with thermal-responsive Gatekeeper for NIR Light-Triggered Chemo/photothermal-Therapy. Small 12, 4286–4298. 10.1002/smll.201601137 27376247

[B22] Li J. Cui D. Huang J. He S. Yang Z. Zhang Y. (2019a). Organic Semiconducting Pro‐nanostimulants for Near‐Infrared Photoactivatable Cancer Immunotherapy. Angew. Chem. Int. Ed. 58, 12680–12687. 10.1002/anie.201906288 31278823

[B23] Li J. Cui D. Jiang Y. Huang J. Cheng P. Pu K. (2019b). Near‐Infrared Photoactivatable Semiconducting Polymer Nanoblockaders for Metastasis‐Inhibited Combination Cancer Therapy. Adv. Mater. 31, 1905091. 10.1002/adma.201905091 31566279

[B24] Li J. Luo Y. Pu K. (2021). Electromagnetic Nanomedicines for Combinational Cancer Immunotherapy. Angew. Chem. Int. Ed. 10.1002/anie.202008386 32671893

[B25] Li J. Pu K. (2019). Development of Organic Semiconducting Materials for Deep-Tissue Optical Imaging, Phototherapy and Photoactivation. Chem. Soc. Rev. 48, 38–71. 10.1039/c8cs00001h 30387803

[B26] Li J. Pu K. (2020). Semiconducting Polymer Nanomaterials as Near-Infrared Photoactivatable Protherapeutics for Cancer. Acc. Chem. Res. 53, 752–762. 10.1021/acs.accounts.9b00569 32027481

[B27] Li J. Yu X. Jiang Y. He S. Zhang Y. Luo Y. (2020a). Second Near‐Infrared Photothermal Semiconducting Polymer Nanoadjuvant for Enhanced Cancer Immunotherapy. Adv. Mater. 33, 2003458. 10.1002/adma.202003458 33325584

[B28] Li Y. Hu J. Liu X. Liu Y. Lv S. Dang J. (2019c). Photodynamic Therapy-Triggered On-Demand Drug Release from ROS-Responsive Core-Cross-Linked Micelles toward Synergistic Anti-cancer Treatment. Nano Res. 12, 999–1008. 10.1007/s12274-019-2330-y

[B29] Li Z. Hu Y. Fu Q. Liu Y. Wang J. Song J. (2020b). NIR/ROS‐Responsive Black Phosphorus QD Vesicles as Immunoadjuvant Carrier for Specific Cancer Photodynamic Immunotherapy. Adv. Funct. Mater. 30, 1905758. 10.1002/adfm.201905758

[B30] Lim S. Park J. Shim M. K. Um W. Yoon H. Y. Ryu J. H. (2019). Recent Advances and Challenges of Repurposing Nanoparticle-Based Drug Delivery Systems to Enhance Cancer Immunotherapy. Theranostics 9, 7906–7923. 10.7150/thno.38425 31695807 PMC6831456

[B31] Lin H. Gao S. Dai C. Chen Y. Shi J. (2017). A Two-Dimensional Biodegradable Niobium Carbide (MXene) for Photothermal Tumor Eradication in NIR-I and NIR-II Biowindows. J. Am. Chem. Soc. 139, 16235–16247. 10.1021/jacs.7b07818 29063760

[B32] Lyu Y. Li J. Pu K. (2019). Second Near‐Infrared Absorbing Agents for Photoacoustic Imaging and Photothermal Therapy. Small Methods 3, 1900553. 10.1002/smtd.201900553

[B33] Maruoka Y. Nagaya T. Sato K. Ogata F. Okuyama S. Choyke P. L. (2018). Near Infrared Photoimmunotherapy with Combined Exposure of External and Interstitial Light Sources. Mol. Pharmaceutics 15, 3634–3641. 10.1021/acs.molpharmaceut.8b00002 PMC7400989 29450993

[B34] Mura S. Nicolas J. Couvreur P. (2013). Stimuli-responsive Nanocarriers for Drug Delivery. Nat. Mater 12, 991–1003. 10.1038/nmat3776 24150417

[B35] Nam J. Son S. Park K. S. Zou W. Shea L. D. Moon J. J. (2019). Cancer Nanomedicine for Combination Cancer Immunotherapy. Nat. Rev. Mater. 4, 398–414. 10.1038/s41578-019-0108-1

[B36] Pardoll D. M. (2012). The Blockade of Immune Checkpoints in Cancer Immunotherapy. Nat. Rev. Cancer 12, 252–264. 10.1038/nrc3239 22437870 PMC4856023

[B37] Pei Q. Hu X. Zheng X. Liu S. Li Y. Jing X. (2018). Light-activatable Red Blood Cell Membrane-Camouflaged Dimeric Prodrug Nanoparticles for Synergistic Photodynamic/chemotherapy. ACS. Nano 12, 1630–1641. 10.1021/acsnano.7b08219 29346736

[B38] Peng J. Yang Q. Xiao Y. Shi K. Liu Q. Hao Y. (2019). Tumor Microenvironment Responsive Drug‐Dye‐Peptide Nanoassembly for Enhanced Tumor‐Targeting, Penetration, and Photo‐Chemo‐Immunotherapy. Adv. Funct. Mater. 29, 1900004. 10.1002/adfm.201900004

[B39] Ramesh A. Kumar S. Brouillard A. Nandi D. Kulkarni A. (2020). A Nitric Oxide (NO) Nanoreporter for Noninvasive Real‐Time Imaging of Macrophage Immunotherapy. Adv. Mater. 32, 2000648. 10.1002/adma.202000648 32390270

[B40] Scheiermann J. Klinman D. M. (2014). Clinical Evaluation of CpG Oligonucleotides as Adjuvants for Vaccines Targeting Infectious Diseases and Cancer. Vaccine 32, 6377–6389. 10.1016/j.vaccine.2014.06.065 24975812 PMC4252359

[B41] Song W. Kuang J. Li C.-X. Zhang M. Zheng D. Zeng X. (2018a). Enhanced Immunotherapy Based on Photodynamic Therapy for Both Primary and Lung Metastasis Tumor Eradication. ACS. Nano 12, 1978–1989. 10.1021/acsnano.7b09112 29420012

[B42] Song W. Shen L. Wang Y. Liu Q. Goodwin T. J. Li J. (2018b). Synergistic and Low Adverse Effect Cancer Immunotherapy by Immunogenic Chemotherapy and Locally Expressed PD-L1 Trap. Nat. Commun. 9, 2237. 10.1038/s41467-018-04605-x 29884866 PMC5993831

[B43] Sun X. Cao Z. Mao K. Wu C. Chen H. Wang J. (2020). Photodynamic Therapy Produces Enhanced Efficacy of Antitumor Immunotherapy by Simultaneously Inducing Intratumoral Release of Sorafenib. Biomaterials 240, 119845. 10.1016/j.biomaterials.2020.119845 32085974

[B44] Tang Y. Li Y. Hu X. Zhao H. Ji Y. Chen L. (2018). “Dual Lock-And-Key”-Controlled Nanoprobes for Ultrahigh Specific Fluorescence Imaging in the Second Near-Infrared Window. Adv. Mater. 30, 1801140. 10.1002/adma.201801140 29920793

[B45] Tao Y. Ju E. Liu Z. Dong K. Ren J. Qu X. (2014). Engineered, Self-Assembled Near-Infrared Photothermal Agents for Combined Tumor Immunotherapy and Chemo-Photothermal Therapy. Biomaterials 35, 6646–6656. 10.1016/j.biomaterials.2014.04.073 24818880

[B46] Triplett T. A. Garrison K. C. Marshall N. Donkor M. Blazeck J. Lamb C. (2018). Reversal of Indoleamine 2,3-Dioxygenase-Mediated Cancer Immune Suppression by Systemic Kynurenine Depletion with a Therapeutic Enzyme. Nat. Biotechnol. 36, 758–764. 10.1038/nbt.4180 30010674 PMC6078800

[B47] Uthaman S. Pillarisetti S. Mathew A. P. Kim Y. Bae W. K. Huh K. M. (2020). Long Circulating Photoactivable Nanomicelles with Tumor Localized Activation and ROS Triggered Self-Accelerating Drug Release for Enhanced Locoregional Chemo-Photodynamic Therapy. Biomaterials 232, 119702. 10.1016/j.biomaterials.2019.119702 31896514

[B48] Wang H. Han X. Dong Z. Xu J. Wang J. Liu Z. (2019). Hyaluronidase with pH‐responsive Dextran Modification as an Adjuvant Nanomedicine for Enhanced Photodynamic‐Immunotherapy of Cancer. Adv. Funct. Mater. 29, 1902440. 10.1002/adfm.201902440

[B49] Wang W. Jin Y. Liu X. Chen F. Zheng X. Liu T. (2021). Endogenous Stimuli-Activatable Nanomedicine for Immune Theranostics for Cancer. Adv. Funct. Mater. 31, 2100386. 10.1002/adfm.202100386

[B50] Xu L. Zhang W. Park H.-B. Kwak M. Oh J. Lee P. C. (2019). Indocyanine green and Poly I: C Containing Thermo-Responsive Liposomes Used in Immune-Photothermal Therapy Prevent Cancer Growth and Metastasis. J. Immunotherap. Cancer 7, 1–14. 10.1186/s40425-019-0702-1 PMC6694491 31412934

[B51] Yang C. Fu Y. Huang C. Hu D. Zhou K. Hao Y. (2020). Chlorin e6 and CRISPR-Cas9 dual-loading system with deep penetration for a synergistic tumoral photodynamic-immunotherapy. Biomaterials 255, 120194. 10.1016/j.biomaterials.2020.120194 32569867

[B52] Yang G. Sun X. Liu J. Feng L. Liu Z. (2016). Light-responsive, Singlet-Oxygen-Triggered On-Demand Drug Release from Photosensitizer-Doped Mesoporous Silica Nanorods for Cancer Combination Therapy. Adv. Funct. Mater. 26, 4722–4732. 10.1002/adfm.201600722

[B53] Zeng Z. Zhang C. Li J. Cui D. Jiang Y. Pu K. (2021). Activatable Polymer Nanoenzymes for Photodynamic Immunometabolic Cancer Therapy. Adv. Mater. 33, 2007247. 10.1002/adma.202007247 33306220

[B54] Zhang C. Pu K. (2020). Molecular and Nanoengineering Approaches towards Activatable Cancer Immunotherapy. Chem. Soc. Rev. 49, 4234–4253. 10.1039/c9cs00773c 32452475

[B55] Zhang N. Song J. Liu Y. Liu M. Zhang L. Sheng D. (2019). Photothermal Therapy Mediated by Phase-Transformation Nanoparticles Facilitates Delivery of Anti-PD1 Antibody and Synergizes with Antitumor Immunotherapy for Melanoma. J. Controlled Release 306, 15–28. 10.1016/j.jconrel.2019.05.036 31132380

[B56] Zhen X. Xie C. Jiang Y. Ai X. Xing B. Pu K. (2018). Semiconducting Photothermal Nanoagonist for Remote-Controlled Specific Cancer Therapy. Nano Lett. 18, 1498–1505. 10.1021/acs.nanolett.7b05292 29342359

